# Maladaptive Plasticity in Aphasia: Brain Activation Maps Underlying Verb Retrieval Errors

**DOI:** 10.1155/2016/4806492

**Published:** 2016-06-27

**Authors:** Kerstin Spielmann, Edith Durand, Karine Marcotte, Ana Inés Ansaldo

**Affiliations:** ^1^Rijndam Rehabilitation Institute, P.O. Box 23181, 3001 KD Rotterdam, Netherlands; ^2^Erasmus MC, University Medical Center Rotterdam, Department of Rehabilitation Medicine, P.O. Box 2040, 3000 CA Rotterdam, Netherlands; ^3^Centre de Recherche de l'Institut Universitaire de Gériatrie de Montréal, 4565 Chemin Queen-Mary, Montréal, QC, Canada H3W 1W5; ^4^École d'Orthophonie et d'Audiologie, Université de Montréal, 7077 Avenue du Parc, Montréal, QC, Canada H3N 1X7

## Abstract

Anomia, or impaired word retrieval, is the most widespread symptom of aphasia, an acquired language impairment secondary to brain damage. In the last decades, functional neuroimaging techniques have enabled studying the neural basis underlying anomia and its recovery. The present study aimed to explore maladaptive plasticity in persistent verb anomia, in three male participants with chronic nonfluent aphasia. Brain activation maps associated with semantic verb paraphasia occurring within an oral picture-naming task were identified with an event-related fMRI paradigm. These maps were compared with those obtained in our previous study examining adaptive plasticity (i.e., successful verb naming) in the same participants. The results show that activation patterns related to semantic verb paraphasia and successful verb naming comprise a number of common areas, contributing to both maladaptive and adaptive neuroplasticity mechanisms. This finding suggests that the segregation of brain areas provides only a partial view of the neural basis of verb anomia and successful verb naming. Therefore, it indicates the importance of network approaches which may better capture the complexity of maladaptive and adaptive neuroplasticity mechanisms in anomia recovery.

## 1. Introduction

Anomia, or impaired word retrieval, is the most prominent and widespread symptom of aphasia, an acquired language impairment that can result from a focal brain lesion [1]. In the context of oral word retrieval, different types of errors (i.e., paraphasia) can occur, including phonemic paraphasia, semantic paraphasia, neologisms, and circumlocutions (i.e., using devious ways to describe words) [2].

The present study focuses on semantic paraphasia in the context of verb retrieval. Verbs carry a critical meaning since they have important functions in the structural formulation of sentences [3]. Therefore, verb paraphasia has a considerable impact on an individual's capacity to convey meaning, which can lead to a substantial handicap. Semantic verb paraphasia occurs when a target verb is replaced by a semantically related verb [4], such as saying “running” instead of “walking.” Research on the cognitive mechanisms underlying the production of semantic paraphasia shows that these may result from impaired phonological processing or impaired semantic processing or a combination of both [5].

Functional neuroimaging techniques allow studying the neural basis underlying verb production and anomia and its recovery. The neural substrate of verb production involves a left frontal cortical network, including the left prefrontal cortex [6], the left superior parietal lobule, the left superior temporal gyrus [7], the left superior frontal gyrus [8], and the primary motor cortex, in the posterior portion of the precentral gyrus [9–11]. In the context of verb anomia, the production of semantic paraphasia may reflect damage of these language-related areas, as well as an attempt to compensate for the impairments resulting from this brain damage as there is a semantic relation between the target and response [12]. This attempt to compensate can be related to the concept of neuroplasticity which refers to a number of brain mechanisms involved in learning and relearning and can be reflected by changes in brain activation patterns highlighted by functional magnetic resonance imaging (fMRI).

Two main forms of neuroplasticity have been studied: functional reactivation, which occurs when previously damaged and inactive areas recover their function after a latency period [13], and functional reorganization, which reflects compensation of the permanent damage of specific brain areas by the recruitment of some other areas not previously involved in language processing [12]. Different types of neuroplasticity may occur during anomia recovery: if this results in functional recovery (as reflected by successful word retrieval), neuroplasticity is defined as adaptive, whereas when errors (such as paraphasia) persist neuroplasticity is considered to be maladaptive [14, 15].

There is an ongoing debate regarding the functional reorganization in anomia recovery and whether these compensatory processes reflect adaptive or maladaptive plasticity. The left cerebral hemisphere (LH) is considered the dominant hemisphere in language processing, at least in right-handed individuals [17]. The fMRI literature has many reports in which LH damage is followed by a shift of language processing to the right cerebral hemisphere (RH), that is, laterality shift [18–21]. However, the extent to which this RH shift reflects adaptive or maladaptive neuroplasticity remains controversial. Some studies focus on the benefits of RH recruitment [22] and emphasize the role of the RH in language processing in healthy subjects [23]. Others suggest that RH recruitment leads to persistent errors, reflecting maladaptive plasticity [24]. Compared to the LH, the RH may have broad overlapping semantic maps: in this case, lexical selection processing would be less semantically specified and would be associated with semantic paraphasia [25]. Another view is that RH recruitment could be beneficial in the short term whereas, in the long term, it could contribute to an incomplete or less efficient improvement compared with a better recovery sustained by the reactivation of LH language processing areas [19–21, 26–28]. Moreover, the extent to which RH recruitment is adaptive or maladaptive may depend on lesion size [12, 27]. These latter authors argue that while minimal damage to core language processing areas leads to maladaptive RH recruitment, extended LH lesions may trigger adaptive RH recruitment by release of the RH potential to process language. Overall, the literature presents a largely negative view on the impact of RH recruitment in the context of aphasia and anomia recovery, in particular in cases of moderate LH damage.

One way of examining the extent of LH and RH recruitment in anomia recovery is by calculating a lateralization index (LI) using fMRI data. The LI reflects hemispheric dominance in terms of the number of activated voxels observed in the context of a specific language task [29]. This index can express the relative contribution of either hemisphere to the processing of specific information, which can be linked to behavioral performance. Several studies have examined the relative contribution of either cerebral hemisphere to anomia recovery within the context of specific and intensive language therapy and by reference to principles of experience-dependent neuroplasticity, derived from animal research [14, 15]. These studies investigated the neurofunctional markers of adaptive plasticity and link right and left hemisphere performance to posttherapy behavior by correlating activation patterns to posttherapy scores on naming tasks [30, 31].

Other studies used noninvasive brain stimulation techniques to modulate cortical excitability in either hemisphere, using repetitive transcranial magnetic stimulation (rTMS) and transcranial direct current stimulation (tDCS). rTMS generates magnetic fields and this can either activate or inhibit neurons. rTMS inhibiting RH areas can significantly reduce speech-error production in nonfluent aphasia [32, 33]. Inhibiting the right pars triangularis (part of the right inferior frontal gyrus) with rTMS improves naming accuracy and decreases naming latency, while activating the right pars opercularis decreases naming accuracy and improves naming latency [33]. With tDCS, a low current can be applied to the brain and, depending on the polarity, it can either enhance (anodal tDCS) or inhibit neural activity (cathodal tDCS) in a certain area. Studies using tDCS mostly combine tDCS with word-finding therapy and find an additional effect of tDCS on naming performance [34, 35]. In summary, rTMS/tDCS studies aim to modulate adaptive plasticity, either by inhibiting RH areas or by enhancing LH areas.

In general, most of the fMRI literature on the recovery from anomia adopts a segregation approach in the analysis of fMRI activation patterns. This is a within-area approach, based on activation changes occurring in isolation [36]. For example, a brain area found to be critical in successful naming is the left Brodmann area 22, which includes the superior temporal gyrus [12, 37]. Another perspective, the integration perspective, gathers brain activation patterns within coherent networks supporting a specific behavior; for example, functional connectivity analysis can be used to study networks of language processing in healthy and brain-damaged populations [38, 39].

In summary, research on the neural basis of anomia recovery has mostly focused on segregating brain areas whose activation is associated either with persistent anomia (i.e., paraphasia), reflecting maladaptive neuroplasticity, or with recovery (i.e., successful naming), reflecting adaptive neuroplasticity. Within this perspective, rTMS/tDCS has been used to modulate RH takeover by inhibiting RH areas, traditionally associated with maladaptive neuroplasticity, or by enhancing LH areas related to adaptive neuroplasticity. However, there is limited knowledge regarding the specific areas whose activation is associated either with the production of paraphasia or with successful naming.

The present study aims to examine maladaptive and adaptive neuroplasticity processes in the context of verb anomia recovery in aphasia. Three participants with nonfluent chronic aphasia were examined in the context of a picture-naming task during event-related fMRI scanning. Activation patterns related to the production of semantic paraphasia were obtained and compared with our previous study that focused on adaptive plasticity, that is, successful verb naming [16]. The relative contribution of the LH and RH to semantic paraphasia and successful naming is explored by calculating an LI.

## 2. Materials and Methods

### 2.1. Experimental Design

The fMRI blood oxygenation level-dependent (BOLD) responses associated with the production of semantic paraphasia produced in the context of verb naming were compared to those related to successful verb naming. BOLD responses were collected in the context of an oral picture-naming verb task within an event-related fMRI paradigm.

### 2.2. Participants

Three male participants from the sample of Marcotte et al. [40], diagnosed with moderate to severe Broca's aphasia, were examined. Inclusion criteria were as follows: (1) a single LH stroke, (2) a diagnosis of moderate to severe aphasia, according to the Montreal-Toulouse battery [41], (3) the presence of anomia in a standardized naming task [42], (4) having French as their mother tongue, and (5) being right-handed prior to the stroke. Exclusion criteria were as follows: (1) the presence of a neurological or psychiatric diagnosis other than stroke, (2) incompatibility with fMRI testing, or (3) a diagnosis of mild cognitive impairment or dementia prior to stroke, based on medical charts, speech-pathology reports, and information from the family. The study was approved by the Ethics Committee of the Regroupement Neuroimagerie/Québec (Canada); all participants provided written informed consent.

**Figure 1:**
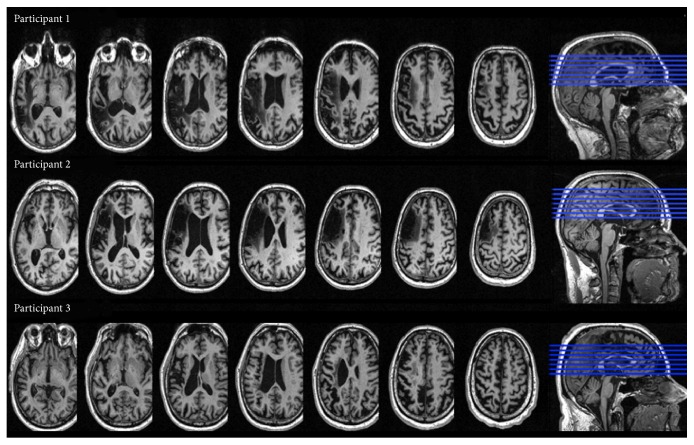
Lesion location for Participant 1, Participant 2, and Participant 3.

Lesion location differed between the participants. Participant 1 (P1) presented a left frontoparietal-temporal lesion, whereas Participants 2 (P2) and 3 (P3) presented a left frontotemporal lesion (Figure 1).

Table 1 presents demographic data; participants were comparable in terms of age and chronic status, and all had extended brain lesions in the left hemisphere (chi-square test: age, *p* = 0.223; months after stroke, *p* = 0.199; years of education, *p* = 0.199; lesion volume, *p* = 0.199).

### 2.3. Procedure

#### 2.3.1. Language Assessment

Aphasia profiles were determined with the Montreal-Toulouse 86 [41]. To ensure stable performance, two baseline naming assessments were obtained before the fMRI study. This baseline assessment was used to select stimuli for the Semantic Feature Analysis therapy, in order to provide personalized therapy (for details, see Marcotte et al. [40]). The selection was done on the basis of individual performance on the Snodgrass and Vanderwart items [42], including object images, and ColorCards® [43], including pictures depicting action verbs.

The present study focused on the ColorCards [43] which included 120 pictures. A total of 80 pictures (60 incorrectly named verbs and 20 correctly named verbs) were selected for the oral picture-naming task during the fMRI session. In addition, 20 digitally distorted images of a subset of these pictures were added as control stimuli.

#### 2.3.2. fMRI Session: Stimuli and Procedure

Participants underwent a practice session in the mock scanner to become accustomed to the scanner noise and environment during the fMRI session. During this session, they were also trained to avoid head movements while naming the stimuli. The stimuli for the picture-naming task (ColorCards) and the control stimuli (i.e., computerized distorted pictures) were projected on a white background by means of a series of mirrors and in a random fashion. Each picture was presented for 4500 ms with an interstimulus interval ranging from 4500 to 8500 ms. Participants were asked to name the pictures representing verbs as accurately as possible, avoiding head movements. In the control condition, participants had to say “BABA” when a computerized distorted picture was presented. Oral and event-related BOLD responses were collected.

#### 2.3.3. Functional Neuroimaging Parameters

Images were acquired using a 3T MRI Siemens Trio scanner, with a standard 8-channel head coil. The image sequence was a T2^*∗*^-weighted pulse sequence (TR = 2200 ms; TE = 30 ms; matrix = 64 × 64 voxels; FOV = 192 mm; flip angle = 90°; slice thickness = 3 mm; acquisition = 36 slides in the axial plane, with a distance factor of 25%, so as to scan the whole brain, including the cerebellum). A high-resolution structural image was obtained before the two functional runs using a 3D T1-weighted pulse sequence (TR = 2300 ms; TE = 2.91 ms; 160 slices; matrix = 256 × 256 mm; voxel size = 1 × 1 × 1 mm; FOV = 256 mm). The protocol was designed in an event-related fashion so that BOLD responses corresponding to each image could be identified.

### 2.4. Data Analysis 

#### 2.4.1. Behavioral and fMRI Data Analysis

Average response times and error rates were calculated for four subtypes of errors: semantic paraphasia, phonological paraphasia, neologism, and circumlocutions. Only semantic paraphasia was produced in a sufficient number to perform fMRI data analysis for all three participants. Therefore, the event-related fMRI responses to semantic paraphasia were analyzed following the same procedures as described by Marcotte et al. [40] and Durand [16]. Activation maps were obtained for each participant by subtracting BOLD responses in the control condition from those obtained in the trials where the answer provided was semantic paraphasia. *t*-tests, performed on each voxel, were considered significant with a cluster size (*k*) ≥ 10 voxels and a *p* value < 0.005. Individual activation maps, including significantly activated brain areas, were determined within the framework of the Talairach atlas [44] and transformed from Talairach space to the spatial coordinates in the Montreal Neurological Institute space [45]. BOLD responses on successful verb naming were examined in our previous study that included the same three participants [16]. In this previous study, BOLD responses in the control condition were subtracted from those obtained in the trials where the answer provided was a correct answer.

Furthermore, an LI [29] was calculated for each participant to estimate the relative contribution of the LH and the RH to the production of semantic paraphasia and successful naming, respectively. Regarding successful naming, data from Durand [16] were used. We applied Lehéricy's algorithm [29], as follows: (LH − RH)/(LH + RH), by which a positive LI corresponds to a LH dominant contribution; strong left lateralization is represented by an LI ranging from 0.5 to 1.0, and weak left lateralization is represented by an LI ranging from 0.25 to 0.5. A negative LI corresponds to a predominant RH contribution; strong right lateralization is represented by an LI ranging from −1.0 to −0.5, and weak right lateralization is represented by an LI ranging from −0.5 to −0.25. An LI ranging from −0.25 to 0.25 represents a symmetric contribution of the left and right hemispheres to processing.

## 3. Results and Discussion

### 3.1. Behavioral Results

Average response times were calculated for paraphasia production; however, due to technical issues these data were not available for analysis. For the 80 pictures, Table 2 presents the error rates and the types of paraphasia produced by each participant during the event-related fMRI study. P1 produced 60 semantic paraphasias and 20 correct responses; P2 produced 15 semantic paraphasias, 32 circumlocutions, and 33 correct responses; and P3 produced 47 semantic paraphasias and 33 correct responses. Only semantic paraphasias were produced in a sufficient number to perform fMRI data analysis for all three participants.

### 3.2. fMRI Results

#### 3.2.1. Single-Subject Brain Activation Maps

Brain activation maps corresponding to maladaptive plasticity, that is, production of semantic paraphasia, in each participant are summarized in Tables 3(a)–3(c). In P1, the production of semantic paraphasia was observed concurrently with significant activation of the precentral gyrus bilaterally, the left superior frontal gyrus (SFG), the inferior frontal gyrus (IFG) bilaterally, the cerebellum (culmen bilaterally, right cerebellar tonsil), the left middle frontal gyrus (MFG), the left brain stem (pons), the left postcentral gyrus, the left fusiform gyrus, the right posterior cingulate cortex, and the right superior temporal gyrus (STG). In P2, the production of semantic paraphasia was observed concurrently with significant activation of the left thalamus (ventral lateral nucleus), the left inferior temporal gyrus (ITG), the cerebellum (left inferior semilunar lobule, right tuber), the right cuneus, the right MFG, the right IFG, the right STG, the right precuneus, the right precentral gyrus, the right middle temporal gyrus (MTG), and the right posterior cingulate cortex. Finally, in P3, the production of semantic paraphasia was observed concurrently with significant activation of the MTG bilaterally, the IFG bilaterally, the left superior parietal lobule, the left inferior parietal lobule, the right precentral gyrus, the right cingulate gyrus, the right SFG, the right putamen, the right MFG, and the right insula.

Table 4 summarizes brain activation maps corresponding to adaptive plasticity (i.e., successful naming) in each participant, adapted from Durand [16]. Successful naming was observed concurrently with significant activation of the MFG bilaterally and the precentral gyrus bilaterally. For the LH, successful naming was observed concurrently with significant activation of the IFG, the SFG, the middle occipital gyrus, the lingual gyrus, the superior parietal lobule, the precuneus, and the pons. For the RH, successful naming was observed concurrently with significant activation of the STG, the MTG, the ITG, the cerebellum (tuber and inferior semilunar lobule), the fusiform gyrus, the sulcus callosomarginalis, and the caudate nucleus.

A comparison was made between brain activation maps associated with semantic paraphasia and those associated with successful naming. In all participants, brain activation maps associated with semantic paraphasia and those associated with successful naming included a number of common significant activation patterns. These common significant activation patterns are highlighted in Tables 3(a)–3(c). In P1, the areas significantly activated with both semantic paraphasia and successful naming included the precentral gyrus bilaterally, the left brainstem (pons), and the right STG. In P2, the areas significantly activated with both semantic paraphasia and successful naming included the cerebellum (tuber), the right STG, and the right MTG. Finally, in P3, the areas significantly activated with both semantic paraphasia and successful naming included the left IFG and the left superior parietal lobule.

#### 3.2.2. Lateralization Indexes

Table 5 presents the LI for the brain activation maps related to maladaptive plasticity (production of semantic paraphasia) and adaptive plasticity (successful naming) for each participant.

The three participants showed bilateral significant activation patterns for both semantic paraphasia and successful naming. Regarding the production of semantic paraphasia, two distinct patterns were observed. Whereas P1 presented a symmetric activation pattern (−0.11), P2 and P3 showed strong predominant LH activation (0.69 and 0.89, resp.). Regarding successful verb naming, P1 showed a symmetric activation pattern (−0.21), P2 showed strong predominant LH activation (0.76), and P3 showed weak predominant LH activation (0.36).

### 3.3. Discussion

The present study aimed to explore maladaptive plasticity, defined as the production of semantic paraphasia, in oral verb naming. Three participants with nonfluent chronic aphasia were examined in the context of a picture-naming task during event-related fMRI scanning. Activation patterns related to the production of semantic paraphasia were obtained and compared to our previous study on adaptive plasticity, that is, successful verb naming [16]. For each participant, the relative contribution of the RH and LH to the production of semantic paraphasia and successful verb naming was determined by calculating an LI.

Results show that the production of semantic paraphasia was associated with the significant activation of right and left hemisphere areas in all three participants. All of these areas are reported to sustain normal language processing in healthy adults [46] and particularly verb production [6–11]. The recruitment of these areas may reflect the attempt to find the correct target verb; however, the attempt to compensate for the system's damaged components is not sufficient and leads to semantic paraphasia that is in some way related to the target word. In addition, the production of semantic paraphasia was associated with specific activation patterns in all participants. This may reflect the impact of individual factors such as lesion location and extension, time elapsed after stroke, age, and education level, all of which have been shown to influence language representation and processing [47–51]. Also, specificities in the mechanisms underlying the production of semantic paraphasia between participants may explain these differences. For example, research on cognitive mechanisms underlying the production of semantic paraphasia shows that these may result from impaired phonological processing or impaired semantic processing or a combination of both impairments [5]. In the present study, we did not examine the degree of relative impairment at either of these processing levels in each participant. Therefore, we cannot exclude the possibility that the mechanisms underlying the production of semantic paraphasia may have differed between participants; this may explain why each participant showed specific activation patterns in relation to the production of paraphasia.

The present study also compared the activation patterns related to the production of semantic paraphasia to our previous study on adaptive plasticity, that is, successful verb naming [16]. In each participant, a number of common activation patterns were observed for semantic paraphasia and successful naming. For P1, these included the precentral gyrus bilaterally, the left brainstem (pons), and the right STG; for P2, these included the right cerebellum (tuber), the right STG, and the right MTG; and for P3, these included the left IFG and the left superior parietal lobule.

Interestingly, also these areas are known for their contribution to language processing in healthy adults and, particularly, sustaining verb production. Some of these areas are known to be involved in lexicosemantic processing. The precentral gyrus is known for its role in action semantics [9–11] and the left precentral gyrus is part of a well-known left-lateralized semantic processing circuit [52–54]. The left IFG is involved in lexicosemantic processing [55] and significant activation of the left superior parietal lobule is related to verb production [7]. Further, the right homologue of the left STG is involved in verb production [7]. Besides these areas involved in lexicosemantics, there are common areas for semantic paraphasia and successful naming that are involved in phonological encoding, articulation, and motor speech. The left IFG and the left STG are involved in phonological processing [56, 57]. The left IFG, left MTG, and cerebellum, together with the primary motor cortex (part of the precentral gyrus), support articulatory planning in speech [22, 57–59]. Regarding the left brainstem (pons) and the cerebellum (tuber), they are part of a cerebrocerebellar loop, sustaining articulation and motor speech stages of word production [60, 61].

The finding that our three participants showed common significant activation patterns during both semantic paraphasia and successful naming may again reflect an attempt of the system to find the correct target verb; sometimes the attempt is successful, and other times it is not. The production of semantic paraphasia may represent a nonefficient system's attempt to compensate for its damaged components, which leads to the selection of error production that is in some way related to the target word. Conversely, successful naming may reflect a function of the spared tissue or an adaptive compensation for the damaged language components, leading to activation of the correct target word. Moreover, the finding of common significant activation patterns during both semantic paraphasia and successful naming also suggests that segregation of brain areas provides only a partial view of the neural basis of verb anomia and successful verb naming and indicates the need to involve network approaches which better capture the complexity of neuroplasticity mechanisms in anomia recovery.

Concerning the lateralization of processing, the contributions of the LH and RH to semantic paraphasia and/or to successful naming are still not totally clear. The LI results of the present study show that both hemispheres contribute to the production of semantic paraphasia and successful naming. RH activation not only is related to the production of semantic paraphasia, but can also be related to successful naming. Therefore, in the present study, RH activation may correspond to efficient compensation in the context of adaptive plasticity processes. This is in line with studies reporting RH activation in the context of successful naming in persons with aphasia [18] and also in healthy participants [23].

The finding that the extent of RH recruitment differed between the three participants might be attributed to lesion size [12, 27]. Larger lesions (associated with poor recovery of language functions) are associated with RH contribution, while in the case of small LH lesions the left perilesional cortex can sustain language recovery. This mechanism is supported by the present data. P1 presents a large lesion and shows a symmetric activation pattern during both semantic paraphasia and successful naming. In contrast, the LI of P2 and P3 reflects predominant LH activation in the presence of smaller LH damage and smaller error rates. The observation of a larger number of semantic verb paraphasia types in P1 can also be related to RH semantic processing abilities. Therefore, it is possible that the RH has access to underspecified semantic representations [25] which may favor the production of semantic paraphasia. However, RH activation in the context of aphasia recovery may reflect the system's attempt to compensate for its damaged components and, to some extent, support access to the correct target word. Therefore, in these three participants, the production of semantic verb paraphasia may reflect an attempt to reach the target in the recovery process.

In summary, these results show that while the global activation pattern differs between the participants, the activation patterns related to maladaptive neuroplasticity and adaptive neuroplasticity comprise a number of common areas. Also, the relative contribution of the left and right hemispheres to maladaptive and adaptive plasticity is not totally clear. This finding challenges the dichotomic distinction between the maladaptive and adaptive roles of the right and left hemispheres, respectively. The present results show that RH recruitment may be associated with adaptive plasticity mechanisms supporting recovery from anomia. Therefore, these findings raise questions regarding the generalizability of rTMS/tDCS studies reporting the advantages of selectively inhibiting the RH homologue of Broca's area to trigger anomia recovery [32–35]. The present findings suggest that inhibiting these areas may, at least in some cases, prevent the expression of the adaptive potential of the RH to support anomia recovery and/or abort the emergence of semantic strategies that may contribute to attenuating the effects of anomia in everyday communication.

The present results support a less dichotomic perspective with regard to the contribution of the right and left hemispheres to recovery from anomia and indicate the importance of adopting a wider perspective when examining the neural basis of anomia recovery. In particular, functional connectivity approaches offer an interesting alternative to the segregation perspective, as they allow considering the dynamic changes that occur within a specific brain network, which may be composed of a similar set of areas. The functional connectivity approach highlights changes in network configuration and activity, depending on a variety of factors, such as complexity level and type of task. Future functional connectivity studies on the neural basis of anomia recovery may help unravel the complex mechanisms underlying neuroplasticity in anomia recovery.

A limitation of the present study is the small number of participants and the fact that all of them were males. However, single-case studies provide important information regarding the variety of idiosyncratic activation patterns in paraphasia and successful naming. Nevertheless, larger samples, including males and females, need to be examined to further elucidate the role of right hemisphere areas and circuits in the adaptive or maladaptive mechanisms that sustain anomia recovery.

## 4. Conclusion

The present study explored maladaptive plasticity in persistent verb anomia by analyzing activation patterns associated with semantic verb paraphasia production in three male participants with chronic nonfluent aphasia. The results show that activation patterns associated with paraphasia production differ across the three participants. This reflects individual factors such as lesion location, time after onset, and the nature of the underlying processing deficits in the context of anomia. The present study also compared the activation patterns related to the production of semantic paraphasia to our previous study on adaptive plasticity, that is, successful verb naming [16]. Interestingly, our three participants showed common significant activation patterns during both semantic paraphasia and successful naming. Finally, the data show that both the LH and the RH are related to the production of semantic paraphasia, thereby questioning the idea of a maladaptive role of the RH. Our findings have implications for future studies aiming at inhibiting or activating specific areas in the context of rTMS/tDCS and suggest that the neural basis of paraphasia and successful naming is not mutually exclusive but may reflect dynamic processes within a relatively limited set of contributing areas.

## Figures and Tables

**Table 1:** Demographic characteristics of the three participants (adapted from Durand [16]).

	Participant 1	Participant 2	Participant 3
Age (years)	67	67	66
Gender	Male	Male	Male
Months after stroke	72	54	241
Years of education	20	15	12
Lesion volume (cm^3^)	167.84	117.84	84.77

**Table 2:** Error rates and the type of paraphasia produced by each participant.

	Participant 1	Participant 2	Participant 3
Semantic paraphasia	60	15	47
Phonological paraphasia	0	0	0
Neologism	0	0	0
Circumlocution	0	32	0

**(a):** 

Left hemisphere							Right hemisphere						
Region	BA	Results SPM			*T*-score	Cluster size	Region	BA	Results SPM			*T*-score	Cluster size
		*X*	*Y*	*Z*					*X*	*Y*	*Z*		
*Frontal lobe, precentral gyrus*	*4*	*−16*	*−28*	*76*	*3.00*	*43*	Limbic lobe, posterior cingulate cortex	29	2	−50	8	3.89	74
Frontal lobe, superior frontal gyrus	6	−20	2	76	2.97	15	Cerebellum, culmen		44	−50	−40	3.74	14
Frontal lobe, inferior frontal gyrus	47	−36	22	−18	2.93	18	Frontal lobe, precentral gyrus	4	66	−2	18	3.70	34
Cerebellum, culmen		−34	−50	−22	4.03	163	Cerebellum, cerebellar tonsil		26	−44	−44	3.47	19
Frontal lobe, middle frontal gyrus	47	−46	40	−12	3.46	38	Frontal lobe, inferior frontal gyrus	47	38	22	−20	3.37	12
Frontal lobe, middle frontal gyrus		−62	10	36	3.07	23	*Temporal lobe, superior temporal gyrus*	*22*	*70*	*−34*	*12*	*5.92*	*332*
*Brainstem, pons*		*−4*	*−22*	*−36*	*4.92*	*32*	*Frontal lobe, precentral gyrus*	*6*	*66*	*−12*	*40*	*4.07*	*155*
Frontal lobe, postcentral gyrus		−58	−10	50	4.72	132							
Occipital lobe, fusiform gyrus		−44	−76	−20	4.10	53							

**(b):** 

Left hemisphere							Right hemisphere						
Region	BA	Results SPM			*T*-score	Cluster size	Region	BA	Results SPM			*T*-score	Cluster size
		*X*	*Y*	*Z*					*X*	*Y*	*Z*		
Thalamus, ventral lateral nucleus		−14	−10	4	3.20	10	Occipital lobe, cuneus	19	18	−86	34	3.15	19
Temporal lobe, inferior temporal gyrus	19	−48	−76	−6	8.64	5520	Frontal lobe, middle frontal gyrus	6	40	0	44	3.11	22
Cerebellum, inferior semilunar lobule		−18	−70	−48	4.66	338	Frontal lobe, inferior frontal gyrus	13	34	16	−22	3.06	33
							Temporal lobe, superior temporal gyrus	22	58	−8	2	2.96	17
							Parietal lobe, precuneus	7	16	−74	56	2.94	11
							Frontal lobe, precentral gyrus	4	16	−36	72	2.93	13
							Temporal lobe, middle temporal gyrus	22	64	−36	2	2.90	23
							Limbic lobe, posterior cingulate cortex		10	−70	12	2.86	22
							Occipital lobe, cuneus	18	12	−86	18	2.85	16
							*Cerebellum, tuber*		*50*	*−56*	*−36*	*5.36*	*679*
							*Temporal lobe, superior temporal gyrus*	*22*	*56*	*12*	*−6*	*5.28*	*141*
							*Temporal lobe, middle temporal gyrus*	*21*	*66*	*−50*	*2*	*4.21*	*91*

**(c):** 

Left hemisphere							Right hemisphere						
Region	BA	Results SPM			*T*-score	Cluster size	Region	BA	Results SPM			*T*-score	Cluster size
		*X*	*Y*	*Z*					*X*	*Y*	*Z*		
Temporal lobe, middle temporal gyrus	39	−62	−60	8	2.90	16	Frontal lobe, precentral gyrus	6	48	0	48	3.28	95
*Frontal lobe, inferior frontal gyrus*	*45*	*−54*	*20*	*18*	*6.90*	*16933*	Limbic lobe, cingulate gyrus	24	6	4	30	3.26	25
*Parietal lobe, superior parietal lobule*	*7*	*−36*	*−72*	*46*	*5.67*	*510*	Frontal lobe, superior frontal gyrus	9	18	48	32	3.10	53
Parietal lobe, inferior parietal lobule	40	−50	−52	48	3.74	169	Lentiform nucleus, putamen		30	2	−10	3.00	25
							Frontal lobe, inferior frontal gyrus	45	64	12	20	2.99	25
							Frontal lobe, middle frontal gyrus		52	34	16	2.92	36
							Insula		38	22	−4	4.40	745
							Temporal lobe, middle temporal gyrus	21	48	8	−40	3.41	11

**Table 4:** Participants 1, 2, and 3: significantly activated areas associated with successful verb naming (adapted from Durand [17]).

	Left hemisphere							Right hemisphere						
	Region	BA	Results SPM			*T*-score	Cluster size	Region	BA	Results SPM			*T*-score	Cluster size
			*X*	*Y*	*Z*					*X*	*Y*	*Z*		
Participant 1	Middle frontal gyrus	6	−38	0	62	4.07	608	Superior frontal gyrus	6	10	2	64	4.54	608
	Precentral gyrus	4	−56	−8	50	4.64	129	Precentral gyrus	6	66	−12	40	4.21	506
	Precentral gyrus	4	−16	−28	72	4.3	111	Middle frontal gyrus	6	28	−6	54	3.98	153
	Pons		−2	−22	−36	4.87	57	Superior temporal gyrus	22	70	−36	12	4.01	64
								Middle temporal gyrus	21	70	−32	4	3.64	64

Participant 2	Middle occipital gyrus	18	−48	−76	−8	7.13	3404	Middle frontal gyrus	6	2	−2	70	6.33	637
	Lingual gyrus	18	−10	−72	−8	6.14	3404	Cerebellum, tuber		50	−56	−36	5.84	83
	Superior parietal lobule	7	−6	−66	60	4.23	116	Fusiform gyrus	37	45	−56	−24	3.9	83
	Precuneus	7	−15	−72	45	3.88	116	Cerebellum, inferior semilunar lobule		12	−70	−48	4.29	74
								Superior temporal gyrus	22	54	14	−6	4.68	54
								Superior frontal gyrus	9	2	52	40	3.91	22
								Middle temporal gyrus	21	66	−50	2	3.8	22

Participant 3	Inferior frontal gyrus	45	−54	22	18	6.05	2028	Sulcus callosomarginalis	8	10	18	48	5.82	1858
	Inferior frontal gyrus	44	−40	10	20	5.84	2028	Middle frontal gyrus	8	6	32	36	4.75	1858
	Middle frontal gyrus	6	−46	12	48	5.25	2028	Middle frontal gyrus	8	36	20	48	3.66	266
	Middle frontal gyrus	6	−22	14	44	4.67	1858	Caudate nucleus		18	−20	22	4.17	205
	Middle frontal gyrus	6	−28	48	18	4.68	449	Inferior temporal gyrus	37	48	−56	−14	4.07	17
	Superior frontal gyrus	9	−8	50	30	3.75	449							
	Superior parietal lobule	7	−36	−72	46	5.17	167							

**Table 5:** Lateralization indexes related to maladaptive plasticity, that is, production of semantic paraphasia, and adaptive plasticity, that is, successful naming, for each participant. A lateralization index ranging from −0.25 to 0.25 represents a symmetric contribution of the left and right hemispheres to processing (participant 1), whereas a positive value indicates a predominant RH contribution to processing (participants 2 and 3) [29].

	Participant 1	Participant 2	Participant 3
Brain activation map for semantic paraphasia	−0.11	0.69	0.89

Brain activation map for successful naming	−0.21	0.76	0.36
